# Association of stress on eating competence in mothers during pregnancy and six months postpartum

**DOI:** 10.1186/s12884-023-06005-6

**Published:** 2023-09-23

**Authors:** Ghasem Pour Sara, Mansoor Ryesa, Akhmadjonova Muzayyana, S. Faith Myles, Lipsky Leah, Nansel Tonja, S. Burger Kyle, Anna Maria Siega-Riz, E. Grace Shearrer

**Affiliations:** 1 Department of Family and Consumer Sciences, University of Wyoming, Laramie, USA; 2 School of Medicine and Health Sciences, George Washington University, Washington, USA; 3 Department of Neuroscience, University of Wyoming, Laramie, USA; 4 Department of Counseling, School, and Educational Psychology, Graduate School of Education, University at Buffalo, The State University of New York, Buffalo, USA; 5 Eunice Kennedy Shriver National Institute of Child Health and Human Development, Bethesda, USA; 6 Department of Nutrition, University of North Carolina Chapel Hill, Chapel Hill, USA; 7 Departments of Nutrition and Biostatistics & Epidemiology, University of Massachusetts Amherst, Amherst, USA

**Keywords:** Pregnancy, Postpartum, Eating competence, BMI, Stress

## Abstract

**Background:**

Perceived stress is related to poor diet quality and unhealthy dietary patterns in women of reproductive age. Eating competence represents a variety of contextual skills reflecting a comfortable and flexible approach to eating and is associated with diet quality and health related behavior. In non-pregnant samples, perceived stress is negatively associated with eating competence. Given that pregnancy and the postpartum period can be periods of high stress, we hypothesized that higher stress in pregnancy would result in lower pregnancy eating competence.

**Methods:**

Women (n = 296, mean BMI_baseline pregnancy_ = 26.3 ± SD 6.0) in the Pregnancy Eating Attributes Study (PEAS) were recruited from the Chapel Hill, North Carolina area. Perceived stress was assessed using the Perceived Stress Scale and eating competence using the ecSatter Inventory at their first trimester and 6-month postpartum visits. We used a mixed effect model to assess the effect of stress by time on eating competence, controlling for baseline pregnancy BMI, race and ethnicity, poverty to income ratio, and WIC status.

**Results:**

Perceived stress was negatively associated with eating competence (b= -0.23, SE = 0.06, p < 0.001). The interaction of stress by time was negatively associated with eating competence (b = -0.15, SE = 0.08, p = 0.03), indicating that the association of stress with eating competence was stronger in postpartum than in pregnancy.

**Conclusions:**

Perceived stress may adversely impact eating competence during both pregnancy and postpartum. Future studies intervening upon stress or eating competence during pregnancy and postpartum may inform potential causal relations.

## Background

Pregnancy and postpartum are periods of novel and changing stress levels and eating behaviors. An estimated 75% of pregnant women experience some degree of stress such as fear of childbirth, unplanned pregnancy, and lack of social support on top of sociodemographic stresses [1]. These stressors evolve in the postpartum period, where women report stress related to changes in relationships, need for social support, changes in body image, and the needs of a new baby [2]. The differences in stressors and overall levels of stress may result in unique changes in eating behavior. Perceived stress, the subjective appraisal of the stressfulness of situations, has been associated with individual eating behaviors such as meal skipping, stress-eating, emotional eating, and excess caloric consumption [3, 4]. Specifically in women, perceived stress is associated with uncontrolled and emotional eating [5, 6]. Similarly, mothers reported increased consumption of comfort foods as a coping mechanism for the stress of COVID-19 lockdown measures [7]. Thus, perceived stress appears to negatively affect multiple individual eating behaviors.

The construct of eating competence encompasses multiple eating behaviors and the biopsychosocial aspects of eating through four domains: eating attitudes, food acceptance, internal regulation of food intake, and management of eating context [8]. Higher eating competence indicates self-confidence in food choices and ability to eat adequate food, openness to new food experiences, and balance between internal and external appetitive signals [9]. Greater eating competence is related to lower emotional eating, uncontrolled eating, and psychological/emotional distress [10]. In non-pregnant samples, greater perceived stress was related to lower eating competence [3, 10, 11], but this relation has not been examined in pregnancy or postpartum, where stressors may uniquely impact eating behaviors.

The present study examined the relationship between perceived stress and eating competence during pregnancy and postpartum. While previous research has offered insights into the complex interplay of stress and eating behaviors [3, 10, 11], the temporal dynamics of this relationship during the pivotal phases of pregnancy and postpartum remain unknown. The present study is the first to evaluate how stress and eating competence change between pregnancy and postpartum. Based on the literature reviewed above, we hypothesized that perceived stress would be negatively associated with eating competence overall. While the primary focus of this study was to investigate the relationship between perceived stress and eating competence during pregnancy and postpartum, it is important to acknowledge the potential influence of maternal BMI on these associations. Maternal BMI during pregnancy can serve as a significant predictor of various maternal and child health outcomes [5, 7], and it may also play a role in shaping maternal eating behaviors and stress responses [6, 10]. Therefore, we also evaluated the role of maternal BMI on the relationship between eating competence and perceived stress. We hypothesized that BMI would be related to decreased eating competence.

## Methods

The data used for this secondary cross-sectional analysis are from the Pregnancy Eating Attributes Study (PEAS), a longitudinal study of neurobehavioral influences on pregnant women’s eating behavior and weight change [12].

### Participants

A total of 458 women were recruited into the PEAS study while receiving prenatal care at University of North Carolina Chapel Hill Healthcare System obstetrics clinics between November 2014 and October 2016. Data collection was completed in June 2018. Participants had to be expecting a singleton pregnancy, at ≤ 12 weeks’ gestation (confirmed at enrollment), have a BMI above 18.5 kg/m^2^, have a delivery at UNC Women’s Hospital, and plan to stay in the area for at least a year after delivery. Exclusion criteria included a history of diabetes, multiple pregnancies, self-reported eating disorder, fetal anomalies, and any medical condition contraindicating participation in the study, such as chronic illnesses or use of effective medications on weight or diet (e.g., cancer, HIV, active renal disease, history of myocardial infarction, thyroid disease, or autoimmune disease). Informed consent was obtained from all the participants and the Informed consent form was approved by the UNC-CH IRB. All research was conducted in accordance with the Declaration of Helsinki.

### Measures

Participants completed in-person clinic visits and online self-reported questionnaires during their pregnancy and postpartum periods. Participants completed 3 visits during pregnancy (visit 1: ≤12 weeks, visit 2: 16–22 weeks, and visit 3: 28–32 weeks’ gestation). The new mothers then completed 3 visits in the postpartum period (visit 4: 4–6 weeks, visit 5: 6 months, and visit 6: 12 months after delivery). Self-report survey measures were completed online within windows associated with each study visit. Women were included in the present analysis if they had complete data for baseline BMI, eating competence and perceived stress measurements (n = 296). Eating competence was assessed at visits 1 and 5, while perceived stress was assessed at visits 1, 3, 4, and 5. For consistency, only the perceived stress measurements at visits 1 and 5 were used in the present analysis to match the eating competence measures. Visit 1 represents the pregnancy timepoint and visit 5 the postpartum timepoint throughout.

### Eating competence

The ecSatter Inventory assesses eating competence in four areas: eating attitudes, food acceptance, internal regulation of food intake, and management of eating context. Responses to the 16 questions are on a Likert scale of always = 3, often = 2, sometimes = 1, rarely = 0; responses are summed for the total eating competence score [13]. Sample items of each of the components are as follows: eating attitude, “I am relaxed about eating”; food acceptance, “I experiment with new food and learn to like it”; food regulation, “I trust myself to eat enough for me”; and contextual skills, “I tune in to food and pay attention to eating.” Eating competence was measured during pregnancy at visit 1 and postpartum at visit 5.

### Perceived stress

The 10-item Perceived Stress Scale (PSS) measures perceived stress, with responses on a Likert scale ranging from 0 (“never”) to 4 (“very often”) [14]. The total score is the sum of items, with higher scores indicating higher perceived stress. Participants completed the PSS during visits 1 and 3, and postpartum visits 4 and 5. Only the values from visits 1 and 5 were used in this analysis for consistency between the pregnancy and postpartum period.

### Anthropometrics

The study staff measured participants’ height at baseline using a stadiometer (to the nearest 0.1 cm) and weight at all visits using a standing scale (to the nearest 0.1 kg) in duplicate. When two measurements differed by more than 1 cm (for height) or 0.2 kg (for weight), a third was taken. BMI was derived by dividing weight in kilograms by the square of height in meters. The final BMI value was the average of the two closest measurements.

### Demographics

Participants self-reported receipt of women infants and children (WIC) benefits, and income to poverty ratio was calculated by dividing the total family income by the appropriate poverty threshold as dictated by household size [15]. Demographic variables were also collected. Race, self-reported by the participant was categorized as white, Black, Asian, American Indian or Native Hawaiian, multi-race, or “other race”. Ethnicity, also self-reported, was categorized as Hispanic or Latino, not Hispanic or Latino, or unknown. Demographic and social-economic status variables were taken at baseline.

### Statistical analyses

All statistical analysis was performed using R version 3.0.6 (R Foundation for Statistical Computing). A linear mixed effects model was fit using the nlme package (version 3.1–140) with a random slope (time) and intercept (subject) to assess the effect of stress by time (pregnancy vs. postpartum) on eating competence. The random slope was set to account for the individual changes in the slope of each participant’s eating competence and stress scores over time from pregnancy to postpartum. Likewise, the random intercept was set to account for differences in individual starting points (intercepts) of eating competence scores. The following covariates were chosen because of their known associations with either perceived stress or eating competence: race and ethnicity [16], poverty to income ratio [17], WIC recipient status (no vs. yes) [18], parity [19], and BMI in pregnancy and postpartum [20].

General Linear Model: Eating Competency ~ Stress*Time Point (pregnancy or postpartum) + BMI + Race + Ethnicity + Household Income + WIC status + parity, random = time/participant.

The linear mixed effects model produces estimates for random effects (the effect of time and participant as random slopes and intercepts, respectively) and fixed effects (the interaction of stress and time, stress, time, BMI, and other covariates). The fixed effects are reported throughout to describe the relationships between eating competence, perceived stress, and time. Statistical significance was set at *p* ≤ 0.05 and the beta value (𝛽) represents the standardized coefficients. Post hoc analyses tested the relationship of stress by time on the individual components within eating competence (eating attitudes, food acceptance, internal regulation of food intake, and management of eating context). The same mixed model described above was used. Additionally, estimated marginal means of linear trends (emmeans package v. 1.6.3) was used to assess differences in the relationship between stress and eating competence (total score and individual components) between pregnancy and postpartum.

## Results

Of the initial enrolled 458 participants, 91 withdrew before delivery, and 46 withdrew during the postpartum period. Participants were between19-41 years of age (mean ± SD age: 30.9 ± 4.26 years). Participants had a mean BMI of 26.29 ± 6.08 at visit 1 and a mean BMI of 26.52 ± 6.34 at visit 5. A majority of the sample identified as non-Hispanic white (67%), with 10% receiving WIC benefits. All participant demographic values can be found in Table 1.

**Table 1:** Demographics by pregnancy and postpartum time points

	Pregnancy	Postpartum
Participants	n = 295	n = 183
Age	30.9± 4.26	
BMI	26.29± 6.08	26.52± 6.34
*Eating Competence*	33.09± 7.53	32.64± 8.03
Eating Attitude	11.08± 2.60	10.96± 2.63
Food Acceptance	4.92± 2.28	5.24± 2.28
Food Regulation	6.58± 1.75	6.42± 1.83
Eating Context	10.51± 2.90	10.03± 2.90
Stress	13.70± 6.18	14.23± 6.50
*WIC Recipient*		
No	83%	
Yes	10%	
Unknown	< 1%	
Not applicable	6%	
Income to poverty ratio	4.23± 1.85	
*Ethnicity*		
Hispanic or Latino	7.1%	
Not Hispanic or Latino	90.2%	
Unknown	2.7%	
*Race*		
White	76.0%	
Black	11.0%	
Asian	5.0%	
American Indian/Native Hawaiian	0.3%	
Multi-race	3.7%	
Other	1.3%	
N/A	2.3%	

Quantitative values are given as mean, standard deviation, and categorical variables are percent out of total participants at baseline (n = 296).

### Association between perceived stress and eating competence from pregnancy to postpartum

**Fig. 1:**
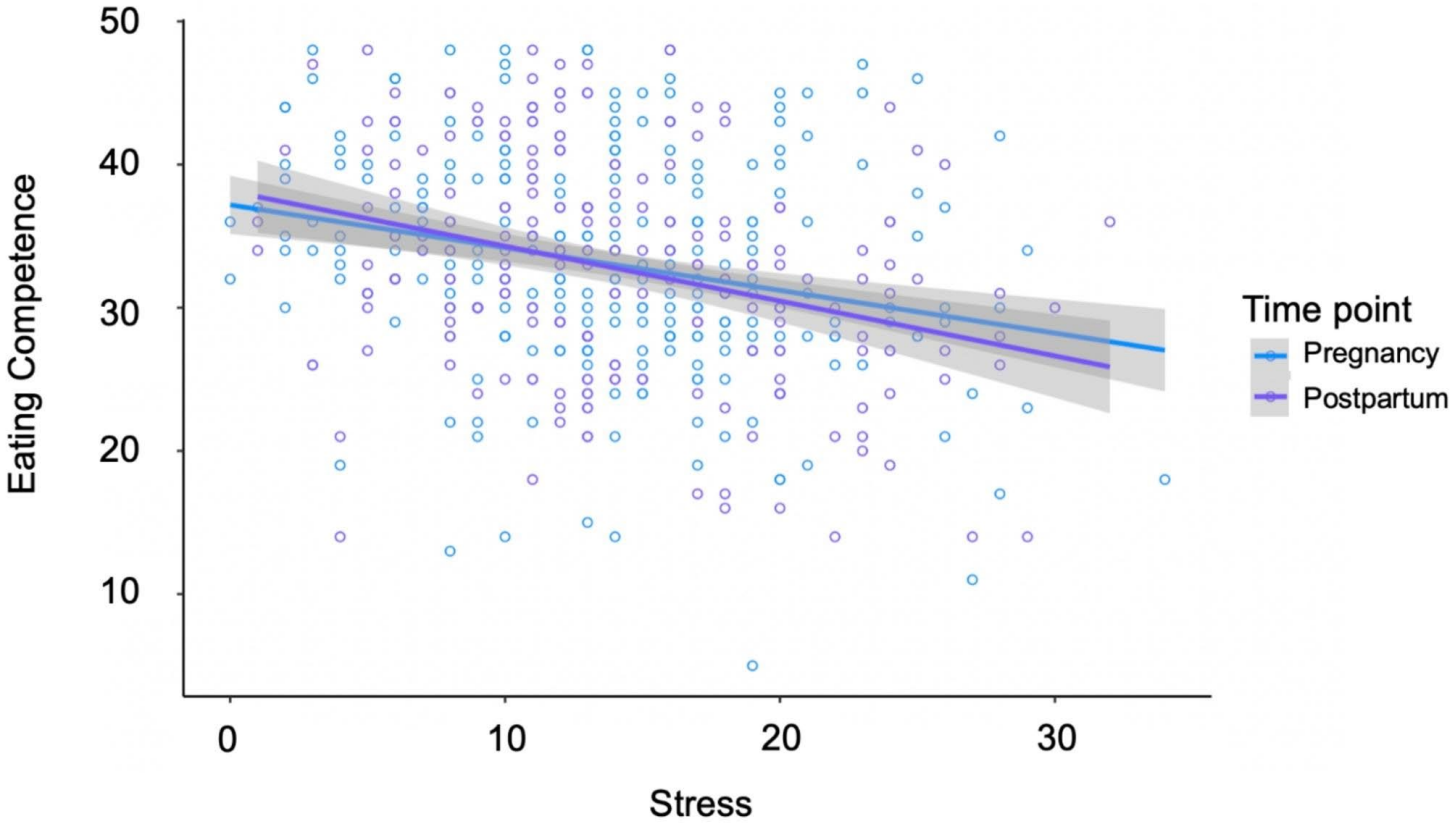
Interaction of time (pregnancy and postpartum) and stress on EC. Stress was inversely related to eating competence in both pregnancy and postpartum (β = -0.22, SE = 0.06, p < 0.001). The interaction of stress and time period was significant (β = -0.17, SE = 0.08, p = 0.03). Pairwise analysis of the slopes shows stress inversely associated with eating competence 54.4% more in the postpartum period compared to pregnancy (b = 0.17, SE = 0.08, p = 0.02)

Perceived stress, across pregnancy and postpartum, was associated with lower eating competence (𝛽 = -0.22, SE = 0.06, p < 0.001, Table 2). The interaction between perceived stress and time (pregnancy and postpartum) was negatively associated with eating competence (𝛽 = -0.17, SE = 0.08, p = 0.03), indicating that perceived stress was negatively associated with eating competence during both time periods – but that the magnitude of the slope differed between time periods (Fig. 1; Table 2). Post hoc analysis of the relationship between eating competence and stress (slope) in pregnancy compared to postpartum confirmed that the magnitude of the slope was greater in postpartum period (𝛽 = 0.17, SE = 0.08, p = 0.03). Maternal BMI was also negatively associated with eating competence (𝛽 = -0.25, SE = 0.07, p < 0.001, Table 2).

**Table 2:** Standardized coefficients (𝛽 values) and p-values from interaction of time and stress on eating competence linear mixed-effects model (n = 296)

	Beta	SE	P
**Time (Pregnancy/Postpartum)**	0.09	1.21	0.055
**Stress**	-0.22	0.06	< 0.001
Stress *Time	-0.13	0.08	0.029
**BMI**	-0.20	0.07	< 0.001
Race^a^			
Black	-0.09	1.53	0.139
Asian	0.05	1.81	0.164
Native American/Native Hawaiian	-0.02	7.52	0.702
Multi-race	0.01	2.13	0.575
Other race	-0.05	3.71	0.569
Ethnicity^b^			
Not Hispanic or Latino	-0.01	1.98	0.908
Ethnicity Unknown	0.04	4.53	0.662
Income to poverty ratio	-0.02	0.27	0.503
WIC Status^c^			
WIC Recipient	-0.01	7.26	0.800
Unknown WIC	0.03	1.97	0.479

(a) Referent group was White; (b) Referent group was Hispanic or Latino; (c) Referent group was Not a WIC recipient

### Post hoc analysis of individual eating competence components

Management of eating context was negatively associated with the interaction of stress by time (𝛽 = -0.07, SE = 0.03, p = 0.02) and was negatively associated with stress regardless of time period (𝛽 = -0.07, SE = 0.02, p = 0.002). Eating attitudes (𝛽 = -0.08, SE = 0.02, p < 0.001) and internal regulation of food intake (𝛽 = -0.04, SE = 0.01, p = 0.001) were both related to stress, but were not different between pregnancy and postpartum. Higher food acceptance was reported in the postpartum period compared to pregnancy (𝛽 = 0.75, SE = 0.35, p = 0.04); however, this was not associated with stress.

## Discussion

We investigated the relationship of perceived stress with eating competence during pregnancy and postpartum in a sample of women from the Chapel Hill, North Carolina area. Perceived stress was negatively associated with eating competence during pregnancy and in the postpartum period as hypothesized, but this relationship was stronger in the postpartum period. Post hoc follow up analyses of the individual components of eating competence suggest that stress and contextual skills are negatively related, and this negative relationship is stronger in the postpartum period.

While previous work has shown that greater stress is related to lower eating competence in general population samples [3, 11], this is the first study to evaluate the relationship of eating competence with perceived stress during pregnancy and postpartum. We found that perceived stress was more strongly related to lower maternal eating competence, particularly contextual skills, in the postpartum period compared to during pregnancy. The transition from pregnancy to the postpartum involves changing roles and identities, physical changes, returning to work, as well as the addition of a childcare duties, leading to increased stress [21]. New mothers report high “overload,” or too many responsibilities for the time available, as a leading postpartum stressor [22]. Despite this, stress management interventions have historically focused on the immediate weeks following childbirth, and little is known about postpartum stress beyond the initial month [23].

The negative association between perceived stress and contextual skills was also stronger in the postpartum period compared to during pregnancy. Previous work in non-pregnant adults showed contextual skills are negatively associated with perceived stress [11]. Contextual skills include meal planning, using nutrition labels, eating outside the home, and meal preparation [9], and are associated with improved adherence to dietary recommendations and increased food diversity [24]. Qualitative work has shown that meal planning is a strategy for healthy cooking during pregnancy, but that the mental energy required for meal planning was a noted barrier [25]. Perceived stress may contribute to this lack of mental energy [26]. Thus, perceived stress may induce mental fatigue, impairing contextual skills and overall decreasing eating competence. More longitudinal work is needed to understand the relationship between contextual skills and perceived stress in the postpartum period. Improving contextual skills, reducing perceived stress, or both may be attractive eating behavior intervention targets in the postpartum period.

The differences seen between pregnancy and postpartum could be in part due to different eating motivations. Focus groups with pregnant women highlight the desire to be healthy for their baby [27, 28]. Healthcare providers similarly focus on health behaviors, such as healthy eating, during pregnancy specifically because it is a finite period and therefore more likely to be adopted [28]. Similarly, Werchan and colleagues hypothesize that the motivation to have a healthy baby may increase engagement in active coping strategies (exercise, eating healthier, increased self-care) [29]. On the other hand, without the incentive of having a healthy pregnancy and with higher tiredness and lack of mental energy in the postpartum period may result in diminished contextual skills and overall poorer eating competence.

Despite differences in eating competence, previous work from this cohort has shown that the dietary intake during pregnancy and postpartum were similar, with slight changes between the two periods regarding meal regularity and eating in the morning [30, 31]. This suggests that women may feel less competent eating but are still able to maintain a diet similar to that in pregnancy. Given that contextual skills are important for meal planning, the changes in meal regularity and eating in the morning could be related to the interaction of stress and contextual skills although this was not directly tested. Longer longitudinal studies into the postpartum period are needed to evaluate if the decline in eating competence precedes changes in diet in relation to perceived stress. Alternatively, the change in eating competence and perceived stress in the postpartum period may be related to alcohol or caffeine consumption. The 2015 healthy eating index (used in this study) does not separately account for alcohol nor caffeine intake and thus may mask differences in diet between pregnancy and postpartum. Given that both alcohol and caffeine can be used as coping mechanisms [32, 33], future research should additionally investigate the relationship between alcohol and caffeine intake with eating competence and perceived stress in the postpartum period.

The present study’s results should be viewed within its strengths and limitations. This is the first study to examine perceived stress and eating competence during pregnancy and postpartum. Assessment of perceived stress and eating competence at multiple time points allowed us to examine differences in associations between pregnancy and postpartum. The sample was more educated and less racially diverse than the US population, though reflective of the geographical area from which it was drawn. Further, the sample was recruited from women obtaining first-trimester prenatal care, which minimizes the generalizability to other areas and women without healthcare access. More research is needed to understand the relationship between eating competence and perceived stress in low-income and traditionally marginalized groups, which likely experience higher and unique stress. Further, given the association between eating competence and stress in the postpartum period, future research should evaluate postpartum depression as a potential mediating factor. Although women in the present study completed the Edinburgh postnatal depression scale, only 12% reported possible depression. Additionally, although our data indicates a difference in the magnitude of relationship between eating competence and stress between pregnancy and postpartum, future studies are needed to assess the clinical and or practical significance. Future work is needed to determine the how changes in maternal eating competence relate to diet quality and excess weight gain. Finally, due to the cross-sectional nature of the analysis causal effects cannot be drawn.

## Conclusions

In summary, perceived stress is negatively associated with eating competence, and particularly contextual skills, during pregnancy and more so in the postpartum period. Reducing perceived stress, improving eating competence, and targeting contextual skills may be useful intervention targets for both pregnant women and those in the postpartum period. Additional longitudinal and experimental research is needed to determine the causal relationship between stress and eating competence.

## Data Availability

Data is available upon request. Please contact Dr. Leah Lipsky.

## Code Availability

Code for the analysis can be found here: https://github.com/m2aenad/EC_STRESS.
